# Invasive squamous-cell carcinoma in Jorge Lobo's disease: Report of two cases and review of the literature

**DOI:** 10.1016/j.mmcr.2022.04.002

**Published:** 2022-05-20

**Authors:** Arival Cardoso de Brito

**Affiliations:** Service of Dermatology, Department of Dermatopathology, Federal University of Para (UFPA), Av. Generalíssimo Deodoro, 805 APT-302, CEP: 66050-160, Nazaré, Belem, Para, Brazil

## Abstract

Jorge Lobo's disease is a chronic granulomatous cutaneous-subcutaneous mycosis caused by the fungus *Lacazia loboi*, seen mainly in tropical and subtropical regions. Malignant transformation rarely occurs in this infection. In the present manuscript, we report two cases of Jorge Lobo's disease complicated by invasive squamous cell carcinoma: a 67-year-old man with a 26 -year history of Jorge Lobo's disease. In this case, the malignant tumor was surgically removed. The second case was a 75-year-old man with 22-year history of mycosis. In both patients the diagnosis of squamous cell carcinoma was confirmed by histopathological examination. There was no an immunocompromised state associated in these patients. The cases, diagnosis, complication, histopathologic findings of this disease are discussed.

## 1 Introduction

Jorge Lobo's disease (JLD) or Lacaziosis is a chronic granulomatous cutaneous-subcutaneous infection, caused by the fungus *Lacazia loboi* which was originally depicted by Jorge Lobo in 1931 in Recife-Pernambuco (Brazil), in a 52-year-old-man, that worked as a rubber collector in Amazonas state [1]. The disease occurs predominantly in several countries of Latin America, seen mainly in the Amazon region, majority in men, aged 20–40 years, especially rubber workers, farmers, fishers, hunters, prospecting for gold, diamonds and others minerals, soldiers in military service, several native tribes and other people with forest activities. JLD is exceptionally found in some patients on other continents. Species bottlenose dolphins - the marine *Tursiops truncatus* and marine-freshwater *Sotalia guianensis* and *S*. *fluviatilis* have been reported infected by fungus *L. loboi* [2,3].

### 1.1 Case 1

**Fig. 1:**
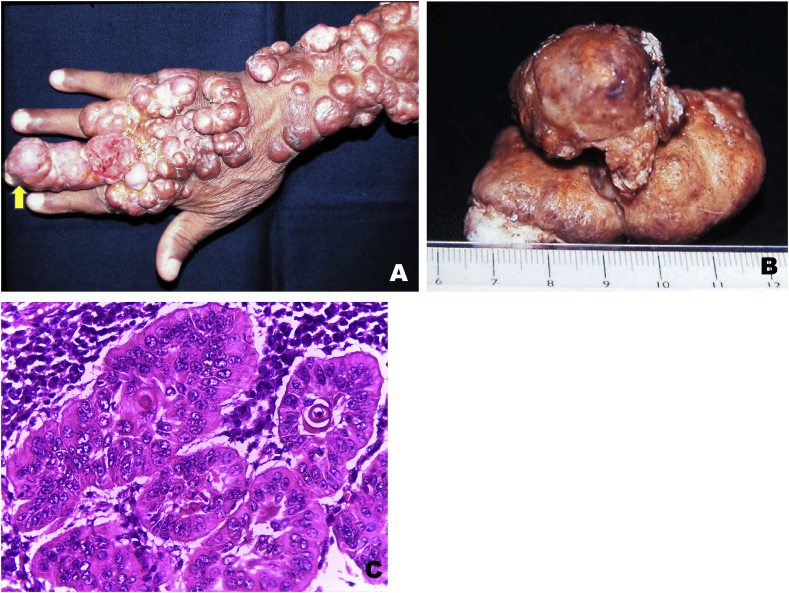
**– Case 1** – **(A)** Tumor on the distal phalanx of the third finger (yellow arrow) and multiple keloid-like nodules on the back of the right hand and forearm ipsilateral. **(B)** Excisional biopsy of the tumor on the distal phalanx of the third finger. **(C)** Invasive well-differentiated squamous-cell carcinoma. H&E x40 magnification.

**Fig. 2:**
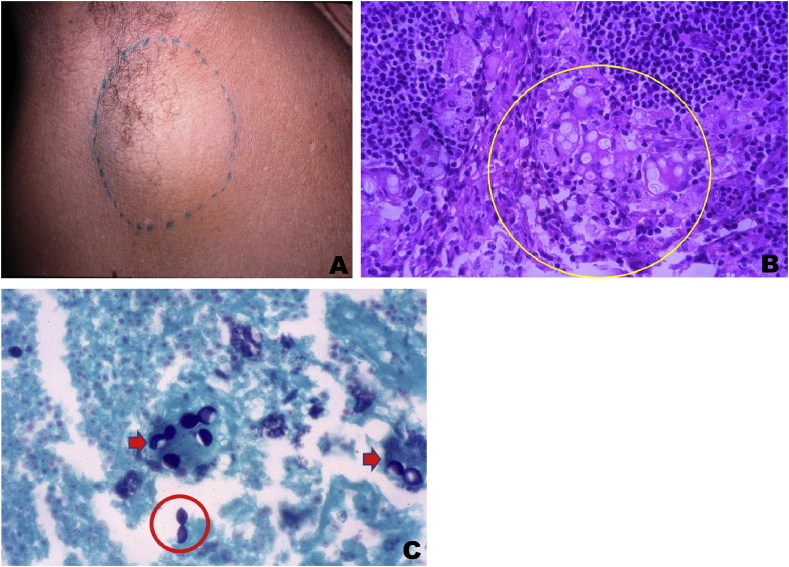
**– Case 1 – (A)** Enlarged lymph nodes in the right axillary circumscribed with a demographic pencil. **(B)** Axillary lymph node: granuloma of macrophages and giant cells containing fungi in paracortical area (yellow circle). H&E x40 magnification. **(C)** Axillary lymph node: several intracellular (red arrows) and extracellular fungal organisms (red circle). Gomori methenamine silver stain. x40 magnification.

A 65-year-old man, originating from state of Para, Brazil, reported a 26-year history of Jorge Lobo's disease with keloid-like nodules on the right arm. He was treated with clofazimine, ketoconazole and itraconazole without benefits. Physical examination showed growth of a nodule two months ago in the distal phalanx of the third finger of the right hand and an enlarged, tender lymph node in the right axillary. An excisional biopsy of the nodule of the third finger and a right axillary lymphadenectomy was performed, both specimens fixed in formalin and embedded in paraffin (Fig. 1A and B). The excised cutaneous specimen was brown, firm consistency and measured 6,2 × 3,6 × 0,6 cm (Fig. 1C). Histological sections of both specimens were stained with hematoxylin and eosin (H&E) and Gomori methenamine silver (GMS). Histopathological analysis of the skin specimen revealed in the dermis tumor arranged in infiltrative islands made up of epithelial cells with pleomorphic and hyperchromatic nuclei, individual cell keratinization, horn pearls and atypical mitotic figures, establishing a diagnosis of squamous cell carcinoma. (Fig. 2A). Microscopic evaluation in one of the 5 examined lymph nodes revealed there were no structural alterations on the capsule and lymphoid follicles. Pronounced sinus histiocytosis and clusters of macrophages with syncytial aspect and giant cells containing several fungi in paracortical area. Hematoxylin and eosin (H&E) and Gomori methenamine silver (GMS). Signs of malignancy were absent (Fig. 2B and C).

### 1.2 Case 2

**Fig. 3:**
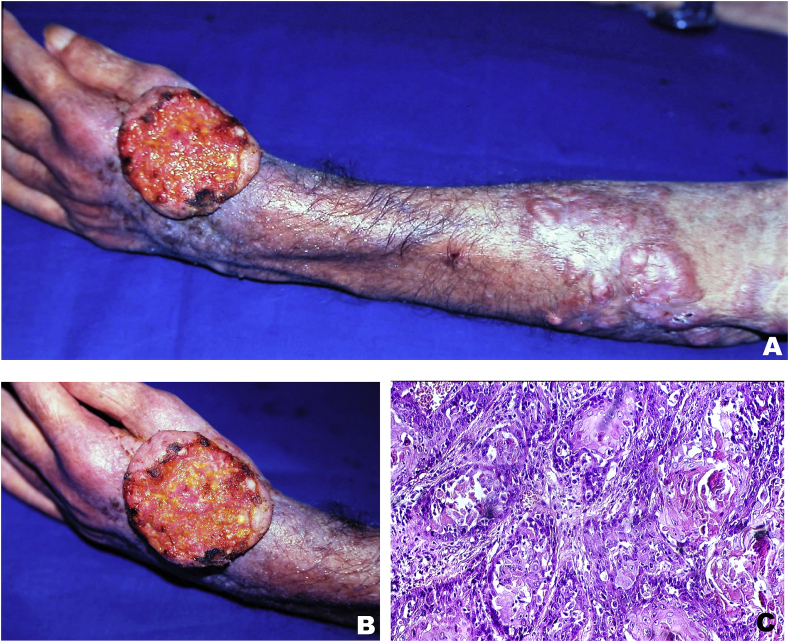
**– Case 2 – (A)** JLD and SCC. Large ulcer on the back of the left hand and plaques/keloid-like nodules on the forearm ipsilateral. **(B)** SCC: prominent ulcer with sharp, raised edges on the back of the left hand. **(C)** Moderately well differentiated SCC. H&E x10 magnification.

A 75-year-old man, originating from state of Para, Brazil, had worked in the rubber plantation for many years. He reported a 22-year history of an asymptomatic papule in the left wrist that evolved into a nodule and over the years similar new lesions appeared on the left forearm. He was treated with several drugs without improvement. Physical examination showed multiple reddish-brown keloid-like nodules and plaques with a smooth surface on the left forearm. On the back of the left hand, large ulcer with raised edges measured about 6.8 cm in major diameter (Fig. 3A and B). There was no lymphatic involvement. Ulcer biopsy was fixed in formalin, embedded in paraffin and histological sections were stained with hematoxylin and eosin (H&E). Microscopic examination showed nests of squamous epithelial cells with abundant eosinophilic cytoplasm, pleomorphic vesicular nuclei, individual cell keratinization and atypical mitotic figures that confirmed the diagnosis of squamous cell carcinoma. (Fig. 3C). This patient did not return to the Dermatology Clinic of the Federal University of Para (UFPA) for treatment of the disease.

## 2 Discussion

JLD is prevalent in Latin and South America – Brazil, Colombia, Suriname, Venezuela, French Guyana, Costa Rica, Panama, Bolivia, Peru, Ecuador, Guyana, Mexico.

It is admitted that *L. loboi* - present in soil, plant and water - penetrates the human skin by single or multiple trauma. JLD is endemic in Amazon area with dense forest, many rivers, humid climate, high temperature, annual rainfall above 2000 mm, environmental conditions that may favor the development of the fungus in soil, plants and water [2,3].

There are about 550 cases in the world including indigenous people. In central Brazil – Xingu Indigenous Park-, the mycosis is prevalent in Kaiabi Indians with 63 cases registered [4].

*Lacazia loboi* is an uncultivated obligate pathogenic fungus, globose, “lemon-shaped” cells, 6–12 μm in diameter, reproducing by simple or multiple budding and multiples a bead-like pattern of 3–10 cells connected by narrow tubes. *Lacazia lobo*i is phylogenetically related to *Paracoccidioides*, *Blastomyces*, *Histoplasma* and *Coccidioides*.

JLD is characterized by the appearance of slowly developing polymorphous lesions: keloid-like nodules, ulcerated or verrucous plaque-like, usually at the site of trauma on exposed areas of the body – ears, face, arms, legs, chest. No cases have been reported in mucosal membranes. Disseminated lesions occur by contiguity, autoinoculation and lymphatic route. Bacterial infection is a very frequent complication.

The diagnosis of fungal infection is established by the clinical appearance of the skin lesions and laboratory tests: direct examination of potassium hydroxide preparations and histopathological examination of biopsy. These examinations reveal numerous globular yeast-like fungi, with a double-contoured refracting membrane, isolated and in chains with several organisms. Negative cultures to *L. loboi*.

In cases of patients with isolated, circumscribed, lesions of JLD, surgery is the best therapeutic approach. Cryosurgery is another therapeutic option in these cases. Drugs used successfully in subcutaneous mycoses and systemic fungal infections - oral azole antifungals, amphotericin B and liposomal amphotericin B, sulfamethoxazole and trimethoprim, clofazimine - are generally unsatisfactory in patients with multiple or disseminated skin lesions. Relapses are very common.

Differential diagnosis-the following should be considered: leprosy; leishmaniasis – mainly diffuse cutaneous leishmaniasis -, sporotrichosis, paracoccidioidomycosis, phaeohyphomycosis, blastomycosis, chromoblastomycosis, histoplasmosis, mycetoma, Kaposi's sarcoma, non-Langerhans cell histiocytoses, cutaneous tuberculosis, benign and malignant tumors, cutaneous metastases.

The records of skin carcinoma from Jorge Lobo's disease lesions are very rare in the literature. Baruzzi et al. [5] recorded four cases of skin carcinoma in Kaiabi Indians: basal cell carcinoma in Indian woman (face) and three cases of SCC in males: one on the penis, one on the back of the upper right arm and one on the left leg. Two Kaiabi Indians who had tumor recurrence died of metastasis. Malignant transformation is reported by Nogueira et al. [6] in a 87-year-old man with disseminated JLD. Microscopic evaluation of the ulcerated lesion confirmed the diagnosis of the SCC and JLD. Another case in an 83-year-old man with disseminated skin lesions of JLD on his limbs is recorded by Lima et al. [7] Histopathological analysis of the left upper limb tumor demonstrated SCC. This patient died. Brito et al. [8] reported a 64-year-old man, agricultural worker, with 30-year history of JLD. The lesions were distributed throughout the skin. Physical examination showed an extensive vegetating plaque on the left leg. Histopathological analysis of the tumor revealed a well-differentiated, ulcerated SCC adjacent to the inflammatory infiltrate with giant cells and abundant fungal yeast compatible with *Lacazia loboi*. Microscopic examination of the left inguinal lymph nodes showed SCC in one of the 13 examined lymph nodes.

The involvement of lymph nodes in JLD has been little studied, in most publications, in clinical and histopathological aspects. It is important to emphasize the evaluation of lymph nodes in this disease, to detect the possibility of recurrence or dissemination of lesions from lymphatic involvement.

Two cases of the lymph node involvement were recorded by Silverie et al., [9] but histopathological examination was performed in only one of them. Destombes et al. [10] describes lymph node enlargement on two patients from French Guyana. Baruzzi et al. [11] recorded a case of lymph node involvement in a Kaiabi Indian with JLD, but there is no information on biopsy and histopathological examination to confirm infection by the fungus. In the two cases reported by Dias et al. [12] one of them presented keloid-like nodules in the right upper limb and an epitrochlear lymph node whose microscopic analysis showed a granuloma with fungi in giant cells. Wiersema (1971) [13] recorded involvement of regional lymph nodes in six patients. The author did not report any histopathological examination of the patient's lymph nodes. A report from Azulay et al. [14] describes a patient with a tumor on the left ear associated with an ipsilateral retroauricular lymph node. Histopathological examination of both specimens confirmed the diagnosis of JLD. Opromolla et al. (2003) [15] reported two cases: case 1, with lesions on the left thigh and a lymph node in the inguinal region. Histological examination of the cutaneous lesion showed granuloma with fungi inside histiocytes and giant cells. The lymph node revealed a chronic lymphadenitis and fragmented fungi stained by methenamine silver. Case 2 showed lesions on the right leg and a lymph node in the inguinal region. Both lesions were excised. This patient returned four years later with new lesions on the right thigh, on the graft and lymph node in the inguinal region. Nodular lesion and several smaller keloid-like lesions on left knee. Histological analysis of the skin lesions revealed granulomas with fungi inside histiocytes and giant cells. The inguinal lymph node showed scattered giant cells containing fungi.

## 3 Authors’ contributions

Arival Cardoso de Brito: Approval of the final version of the manuscript; design and planning of the study; acquisition of case history; preparing the histologic pictures with legends; effective participation in research orientation; drafting and editing of the manuscript; intellectual participation in the propaedeutic and/or therapeutic conduct of the studied cases; critical review of the literature; critical review of the manuscript.

## Funding source

There are none.

## Consent

Written informed consent was obtained from the patient or legal guardian(s) for publication of this case report and accompanying images.

## Notes

1.1- Clinical photographs captured by a Nikon Coolpix P520 camera. 1.2- Photomicrographs captured by Leica DM500 photomicroscope.

## Declaration of competing interest

There are none.
